# Computed Tomography Perfusion is a Useful Adjunct to Computed Tomography Angiography in the Diagnosis of Brain Death

**DOI:** 10.1007/s00062-017-0631-7

**Published:** 2017-11-17

**Authors:** M. Sawicki, J. Sołek-Pastuszka, K. Chamier-Ciemińska, A. Walecka, J. Walecki, R. Bohatyrewicz

**Affiliations:** 1 Dept. of Diagnostic Imaging and Interventional Radiology, Pomeranian Medical University, Szczecin, Poland; 2 Clinic of Anesthesiology and Intensive Care, Pomeranian Medical University, Clinical Hospital No1, Unii Lubelskiej 1, 71252 Szczecin, Poland; 3 The Centre of Postgraduate Medical Education, Warsaw, Poland

**Keywords:** Brain death, Four-dimensional computed tomography, Multidetector computed tomography, Perfusion imaging

## Abstract

**Background:**

In the diagnosis of brain death (BD), computed tomography angiography (CTA) results in some cases show intracranial filling, leading to diagnostic confusion. Because cerebral circulatory arrest commences at the capillary level, we hypothesized that computed tomography perfusion (CTP) would be a more sensitive approach than CTA; therefore, the aim of the study was to compare the sensitivities of CTP and CTA in the diagnosis of BD.

**Material and Methods:**

Whole brain CTP was performed in patients in the intensive care unit diagnosed with BD and CTA was derived from CTP datasets. Cerebral blood flow (CBF) and volume (CBV) were calculated in all brain regions. The CTP findings were interpreted as being consistent with a diagnosis of BD (positive) when CBF and CBV in all regions of interest (ROIs) were below 10 ml/100 g/min and 1.0 ml/100 g, respectively. The CTA findings were interpreted using a 4-point grading system.

**Results:**

A total of 50 patients were included in the study. The CTP results revealed CBF from 0.00 to 9.98 ml/100 g/min (mean, 1.98 ± 1.68 ml/100 g/min) and CBV from 0.00 to 0.99 ml/100 g (mean, 0.14 ± 0.12 ml/100 g) and were thus interpreted as positive in all 50 patients. In contrast, the CTA results suggested 7 negative cases, providing a sensitivity of 86%. The difference between the CTP and CTA sensitivity results for the diagnosis of BD was statistically significant (*p* = 0.006).

**Conclusion:**

Whole brain CTP may potentially be a feasible and highly sensitive test for diagnosing BD: therefore, performing CTP in combination with CTA in cases when CTA results are negative for BD could increase the sensitivity of CTA.

## Background

Brain death (BD) diagnosis first relies on a clinical examination and the study of brainstem function [1]; however, there are some situations in which clinical examinations are unreliable or cannot be completed because of confounding factors (e. g. neurodepressive agents, metabolic disorder, facial or brainstem damage, infants and children). In these specific situations ancillary tests, particularly documenting cerebral circulatory arrest can be performed to assist the clinician. Moreover, in all cases of suspected BD, the use of instrumental examinations allows the duration of the observation period to be decreased and diagnostic confidence to be increased.

In recent years computed tomography angiography (CTA) became the most commonly used ancillary test for the determination of BD; however, radiologists sometimes become confused when interpreting CTA findings about whether to confirm or reject a diagnosis of BD in deeply comatose patients with areflexia. This diagnostic confusion is caused by the preserved filling of the cortical branches of the middle cerebral artery (MCA), the internal cerebral veins (ICV), or both, and this preservation is observed in 15–16% of brain dead patients, according to recent meta-analyses [2, 3]; however, the phenomenon of persistent intracranial opacification, also known as stasis filling, does not necessarily preclude the diagnosis of BD, as was shown by Sawicki et al. [4]. This hypothesis was supported by Shankar and Vandorpe, who found computed tomography perfusion (CTP) parameters consistent with nonviable brainstem in patients with preserved filling of supratentorial vessels when using CTA [5]. The advent of CT systems with sufficiently wide range in the z‑axis in dynamic scanning has enabled the evaluation of whole brain perfusion with simultaneous assessment of vascular filling with CTP-derived CTA.

Because cerebral circulatory arrest commences at the capillary level, we hypothesized that the addition of whole brain CTP to the commonly used CTA approach would reduce the frequency of negative findings obtained using CTA alone, therefore increasing the sensitivity of the test for the confirmation of BD. Thus, the present study was designed to compare the sensitivities of whole brain CTP and CTA in the diagnosis of BD.

## Material and Methods

### Study Design

**Fig. 1:**
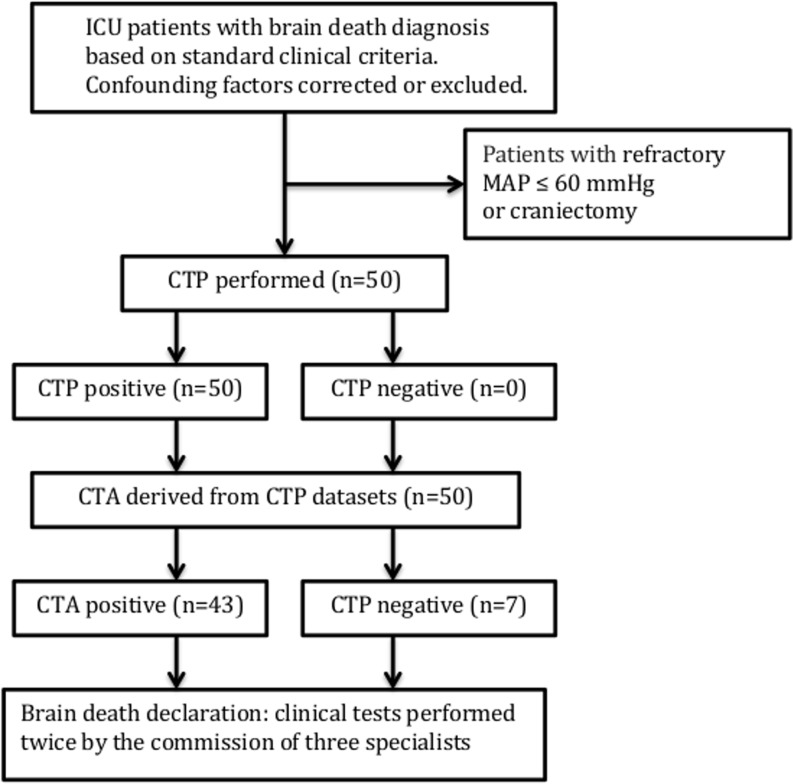
Study flow diagram (*CTP* computed tomography perfusion, *CTA* computed tomography angiography, *MAP* mean arterial blood pressure)

The study participants were recruited from consecutive patients admitted to the intensive care unit of our university hospital (tertiary center) with a diagnosis of BD based on the following standard clinical criteria: deep, unresponsive coma of established etiology capable of causing neurological death, essential artificial ventilation, absence of brainstem reflexes, including pupil, oculocephalic, oculovestibular, corneal, pharyngeal and tracheal reflexes. Confounding factors, including neurodepressant drugs, hypothermia, ongoing hypoxia, and severe endocrine disorders, were corrected or excluded. We excluded patients with refractory mean arterial blood pressure (MAP) ≤60 mm Hg or who had undergone craniectomy, the latter because of the previously shown predisposition of brain dead patients with craniectomy to show preserved intracranial filling in CTA [6]. This predisposition limits the application of CTA in the diagnosis of BD. After receiving a diagnosis of BD, all participants underwent volume brain scanning using CTP and CTA. Shortly after radiological examinations, all patients were declared brain dead. This declaration was made after declaration of coma and determination of brainstem areflexia and absence of respiratory drive using a 10-min apnea criterion. In concordance with the national legal regulations, these tests were performed twice by three specialists. The study flowchart is presented in Fig. 1. Transplantable organs were procured from most of the brain dead patients. In some cases, organ donation was abandoned for medical reasons or because of a lack of consent from family members. In such situations, all forms of life support, including ventilation, were withdrawn and patients underwent cardiopulmonary collapse within a short time.

### CT Data Acquisition

Data were acquired using a 128-slice Siemens Definition AS+ CT scanner (Siemens Healthcare, Erlangen, Germany) after administration of iodinated contrast material (50 ml; Iomeron 400, Bracco Imaging, Konstanz, Germany) at a rate of 6 mLls via a power injector through an 18-gauge intravenous line, followed by saline (30 ml) administered at the same rate. The scanning parameters were 80 kVp and 200 mA. Scans were performed every 1.5 s for 60 s and were started with a delay of 4 s after contrast material injection, providing a total of 40 volume datasets. The total coverage in the z‑axis was 96 mm, with a slice width of 10 mm obtained in 5 mm increments using a shuttle mode (adaptive 4‑D spiral). The patient’s chin was tilted toward the chest to shorten the z‑axis distance between the skull base and the vertex. This tucked position enabled the investigator to visualize the whole brain from the level of foramen magnum to the vertex.

### Postprocessing

#### CTP

Perfusion parameters were calculated using the commercial perfusion software package syngo.via(R) CT Neuro Perfusion Version 2012B (Siemens Healthcare) based on a deconvolution algorithm with least mean square fitting. Processing was performed semiautomatically with the default settings used in routine clinical practice. The use of automatic motion correction was necessary to eliminate possible artifacts caused by ventilation and to-and-fro (shuttle) movement of the table during acquisition. Additional noise was removed using an automatic 4‑D noise reduction tool to improve the quality of the image. An arterial input function (AIF) and a reference vessel were chosen from time-averaged maximum intensity projection (MIP) images. The AIF was manually set to the cavernous segment of the internal carotid artery (ICA). To eliminate partial volume averaging, the original AIF was corrected to the maximal enhancement measured in the reference vessel. An intracranial artery or vein with the highest peak enhancement was selected to serve as the reference vessel (maximal enhancement reference). Major vessels were removed by relative “thresholding” applied to the maximum enhancement. Relative thresholds were set at 8% of the maximum enhancement. A normalization step, routinely used in clinical practice, was not performed in the present study because neither of the cerebral hemispheres could be used as a reference point for normal perfusion.

To determine perfusion values from different brain regions, 1‑cm^2^ circular regions of interest (ROIs) circumscribing the brainstem, including the midbrain (*n* = 2), pons (2), and medulla oblongata (2) as well as the cerebellum (8), cortical regions of the frontal (12), parietal (12), temporal (12), and occipital lobes (8), and the basal ganglia (8), were drawn bilaterally and placed on each 10-mm axial slice. This resulted in a total of 66 ROIs for each patient. Postprocessing, including selection of ROIs, was performed by a board-certified radiologist with over 10-years experience in neuroradiology who was blinded to the results of clinical tests.

#### CTA

The CTA images were automatically reconstructed from the CT perfusion source images using syngo.via(R) CT Dynamic Angio Version 2012B (Siemens Healthcare) as timing-invariant (TI)-CTA. The TI-CTA provides angiography by overlapping all time frames and displaying maximum enhancement over time. Because of a choice of the temporal maximum, this technique is timing invariant, which means that the maximum enhancement of a vessel is displayed independent of its contrast arrival time. Therefore, TI-CTA is not sensitive to delayed arrival of contrast material in cerebral vessels, and thus should display any vessel present. This technique was previously described and shown to be reliable by Smit et al. [7].

### Image Analysis

#### CTP

The CTP findings were interpreted as consistent with BD diagnosis (i. e. positive) when cerebral blood flow (CBF) and volume (CBV) in all 66 ROIs were below the well-established thresholds for neuronal necrosis, i.e. 10 ml/100 g/min and 1.0 ml/100 g, respectively.

#### CTA

The CTA data were derived from CTP datasets as TI-CTA. The images were analyzed first for the appearance and disappearance of contrast medium in the superficial temporal artery branches to confirm that the contrast was injected properly and to eliminate the potential influence of hemodynamic perturbations. The presence of contrast in the different segments of the intracranial arteries was analyzed using CTA on a 4-point scale based on the lack of opacification of the cortical segments of the MCA and the two ICVs. A score of 1 was given for each of the non-opacified vessel segments. The CTA findings were interpreted as consistent with a BD diagnosis (i. e. positive) if the examination revealed bilateral non-filling of cortical segments of the MCA and bilateral non-filling of the ICV. This 4‑point grading system was proposed by Leclerc et al. in 2006 [8]. A neuroradiologist with 10 years of experience in interpreting cerebral CTA and blinded to the results of clinical tests evaluated all CTA images.

### Data Collection

The following demographic and clinical data were collected: age, sex, and cause of brain injury categorized as vascular (ischemic stroke, non-traumatic intracranial hemorrhage) or traumatic or anoxic-ischemic injury. For each CTP ROI, the following parameters were recorded: CBF, CBV, and contrast-to-noise ratio (CNR). The CNR was calculated using the formula CNR = (peak HU mean − baseline HU mean)/baseline HU SD, where HU represents Hounsfield units and SD represents standard deviation. Each CTA opacification of the cortical branches of the MCA and ICV was noted bilaterally.

### Statistical Analysis

The χ^2^-test was used to compare the sensitivities of CTP and CTA in the diagnosis of BD. A value of *p* < 0.05 was considered statistically significant. Statistica 12 software (StatSoft, Tulsa, OK) was used for statistical analyses. A biomedical statistician reviewed the manuscript for clarity of statistical analyses and data presentation.

## Results

A total of 50 patients (27 females, 23 males) with a mean age of 55 ± 18 years (range, 17–78 years) were enrolled in the study. We excluded 6 patients because they had undergone craniectomy. No patients were excluded due to refractory MAP ≤60 mm Hg. Of the enrolled 50 patients, the cause of brain injury was vascular in 36 (72%), traumatic in 5 (10%), and anoxic ischemic in 9 (18%) patients.

In all CTP studies, manual positioning of an ROI onto the cavernous segment of the ICA provided a tissue attenuation curve sufficient for use as the AIF. The mean value of maximum enhancement of the cavernous segment of the ICA was 346 ± 132 HU (range, 120–638 HU). This was the only possible choice as none of the remaining arteries showed satisfactory enhancement. In all cases, this same vessel (i. e. the cavernous segment of the ICA that was used as the AIF) was used as a surrogate of venous output function (VOF). The intracranial veins, including the routinely used superior sagittal sinus, torcular Herophili or transverse sinus, could not be used for the venous output function because they showed vestigial enhancement or entirely lacked enhancement (maximum enhancement was below 100 HU).

**Fig. 2:**
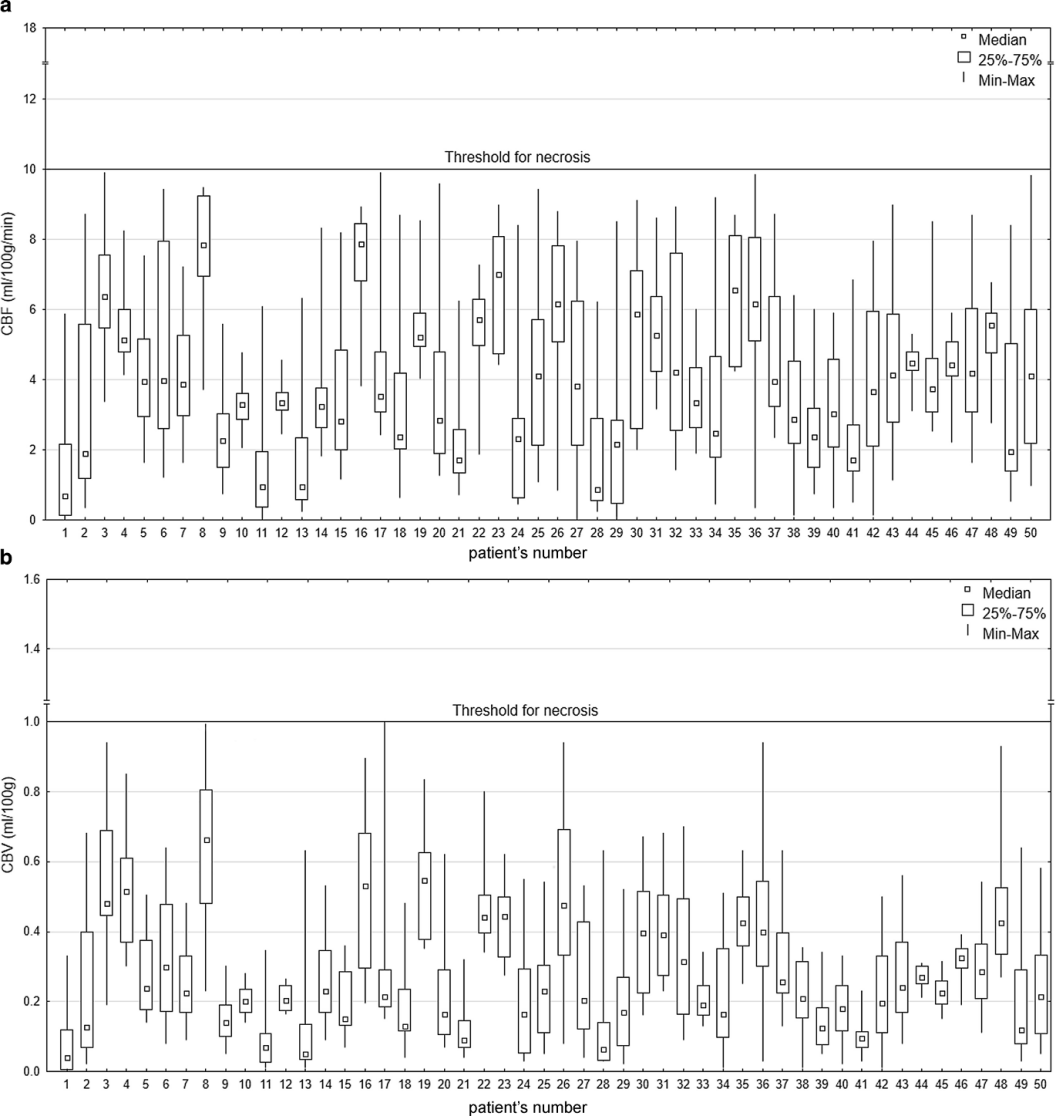
The distribution of CBF (**a**) and CBV (**b**) in 50 patients diagnosed with BD. Box plots present values obtained from 66 ROIs for each patient covering all brain regions. Data are presented as medians, 25–75% interquartile ranges, minimums and maximums. In all cases, the perfusion values are below the thresholds for nonviable tissue

**Fig. 3:**
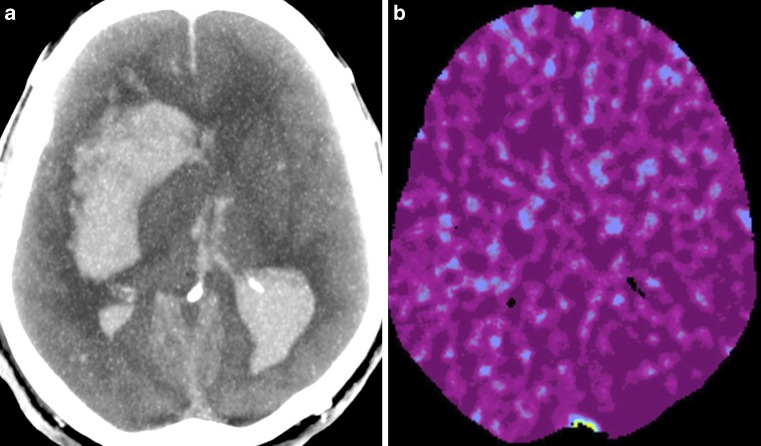
Results of CTA (**a**) and CTP (**b**) in the patient diagnosed with brain death. The CTA shows absence of filling of intracranial arteries and veins and was classified as positive, i. e. consistent with the diagnosis of BD. CTP reveals perfusion values below the thresholds for non-viable tissue and, like CTA, was interpreted as positive, i. e. confirming the diagnosis of BD

The CTP findings revealed CBF from 0.00 to 9.98 ml/100 g/min (mean, 1.98 ± 1.68 ml/100 g/min) and CBV from 0.00 to 0.99 ml/100 g (mean, 0.14 ± 0.12 ml/100 g). In all 50 patients, values in all ROIs were below the thresholds for nonviable tissue, which are 10 ml/100 g/min for CBF and 1.0 ml/100 g for CBV. Therefore, we interpreted the CTP findings in all patients as positive, i.e. the results confirmed the diagnosis of BD. This resulted in a sensitivity of 100% (95% CI, 0.91–1.00) for CTP in the diagnosis of BD. The values of the perfusion parameters for individual patients are presented in Fig. 2. Results in one of the patients in whom CTP and CTA consistently confirmed the diagnosis of BD are presented in Fig. 3.

**Fig. 4:**
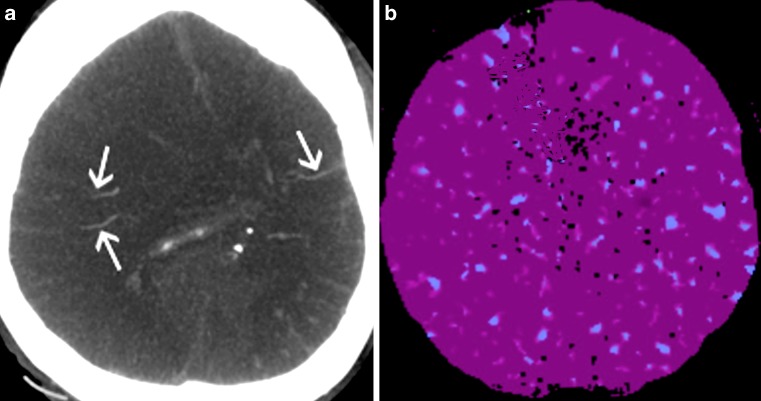
Results of CTA (**a**) and CTP (**b**) in the patient diagnosed with brain death. CTA shows filling of cortical branches of the right and left MCA (*arrows*) and was classified as negative, i. e. inconsistent with the diagnosis of BD. CTP reveals perfusion values below the thresholds for non-viable tissue and, contrary to CTA was interpreted as positive, i. e. consistent with the diagnosis of BD

The use of TI-CTA showed 7 negative results because preserved cortical or deep venous filling was noted, which is inconsistent with the diagnosis of BD (see Table 1). Results in one of these patients are presented in Fig. 4. This resulted in a sensitivity of 86% (95% CI, 0.73–0.94) for CTA in the diagnosis of BD.

**Table 1:** Patient characteristics and imaging findings in cases of false negative CTA results

#	Sex	Age (years)	Cause of brain injury	CTA opacification				BD diagnosis
				MCA-M4 right	MCA-M4 left	ICV right	ICV left	
5	M	56	Vasc	0	1	1	1	Negative
8	M	71	Vasc	0	0	0	1	Negative
17	M	73	tbi	1	0	1	1	Negative
23	F	34	Vasc	0	0	1	1	Negative
29	M	50	Vasc	0	1	1	1	Negative
41	M	44	Vasc	0	0	1	1	Negative
47	M	51	Vasc	1	0	1	1	Negative

*vasc* ischemic stroke and non-traumatic intracranial hemorrhage, *tbi* traumatic brain injury, *BD* brain death, *CTA* computed tomography angiography, *MCA* middle cerebral artery, *ICV* internal cerebral vein, *#* patient’s number

We found a statistically significant difference between the sensitivities of CTP and CTA in the diagnosis of BD (*p* = 0.006; χ^2^-test). The evaluation of image quality showed a mean CNR of 0.34 ± 0.21 (range, 0.09–0.85).

## Discussion

The complete and irreversible failure of central nervous system functions constitutes the authentic frontier between life and death of human beings; however, not all medical schools accept the same concept of BD. Consequently, the criteria for diagnosis are different according to the concept of BD used. The whole BD concept is the most widespread concept, and it is characterized by the irreversible cessation of hemispheric and brainstem neurological functions. In 1976 the Conference of Medical Royal Colleges and their Faculties in the United Kingdom published a statement on the diagnosis of BD defined as the complete, irreversible loss of brainstem function, which pointed to the brainstem as the center of brain function (brainstem death) [9]. This brainstem death concept, in place of the concept of whole brain death, explains why, in some countries, complementary tests are not legally required for the confirmation of clinical BD diagnosis, based upon cessation of brainstem function.

Whatever the adopted concept is, brainstem death or whole brain death, the first step remains the clinical assessment of permanent BD. Nonetheless, some national guidelines state that ancillary tests that confirm irreversible cerebral circulatory arrest can be used as an appropriate tool for the decision on when neurologic examination can be done for the clinical assessment of permanent BD (independently of residual interactions caused by sedative drugs etc.).

Widely accepted ancillary tests include the following: cerebral catheter angiography, perfusion scintigraphy and transcranial Doppler ultrasonography. Recently, CTA was introduced into the diagnosis of cerebral circulatory arrest; however, the main obstacle in the application of CTA is a low sensitivity caused by filling of cortical arteries and/or deep cerebral veins observed in some brain dead patients.

Our study showed that CTP findings revealed whole brain nonviability, as defined as CBF below 10 ml/100 g/min and CBV below 1.0 ml/100 g, in all brain dead patients, including those in whom the results of CTA showed preserved intracranial filling.

Our findings parallel previous research by Sawicki et al. [4] and by Shankar and Vandorpe [5] who revealed that preserved intracranial opacification, also known as stasis filling, can be observed in cases in which CTP shows nonviability of the brain, and thus stasis filling does not preclude the diagnosis of BD. This apparent discrepancy may be explained by a known phenomenon that cerebral circulatory arrest commences in vessels with the slowest blood flow (i. e. the capillaries), which are more susceptible to intracranial hypertension than large and medium size vessels. Therefore, during gradual increase of intracranial pressure, when capillary flow as reflected by CTP has already ceased, a column of contrast medium can still propagate for some time, filling precapillary vessels in CTA. Thus, this phenomenon has negative impact on the sensitivity of the method. A meta-analysis published in the Cochrane database of systematic reviews showed a sensitivity of 85% (mean of 8 included studies) for CTA in the diagnosis of BD [2]. We achieved a similarly low sensitivity of 86% using this approach although, based on our findings, performing CTP in combination with CTA in negative CTA cases could significantly increase the sensitivity of the test; however, recommending CTP in combination with CTA as a mandatory or routine confirmatory test in all cases for the diagnosis of BD would be unreasonable because CT scanners capable of performing whole brain CTP are not currently ubiquitously available.

The current study includes several advances over previous studies. First, we assessed the whole brain using CTP, whereas Sawicki et al. scanned approximately 3 cm in the z‑axis, and Shankar et al. evaluated only the brainstem and provided no absolute values of CBF or CBV [4, 5]. In addition, ours is the first study to use TI-CTA to diagnose BD. This approach enables the detection of intracranial opacification regardless of a delay in contrast medium arrival. In our study, the images obtained from the 4–60 s after contrast medium injection were compiled into a single image. Thus, the use of TI-CTA avoids the problem of having to select a proper delay for CTA scanning after contrast medium injection in the diagnosis of BD. This issue is still debated, and no consensus has been reached so far [10]. Different approaches are proposed, for example, according to current French guidelines, intracranial filling is assessed 60 s after contrast medium injection, whereas in German national regulations it is assessed in the arterial phase [11–13]. Moreover, TI-CTA can be derived from CTP datasets (as in this study); thus, both tests can be performed following a single injection of contrast. Although it is thought that CTP findings in brain dead patients should indicate values of zero for CBF and CBV, the results of the present study did not confirm this assumption, because CBF and CBV values were found to be slightly higher than zero. The ability of CTP to differentiate between extremely reduced blood flow and the absence of blood flow has been studied by Uwano et al. using digital phantoms simulating CBF of 0–2.4 ml/100 g/min and CBV of 0–0.16 ml/100 g [14]. Using different perfusion software packages, they discovered that CBF and CBV values for true zero phantom flow were actually slightly higher than zero. These results and those of the present study suggest that the analysis precision of tissue attenuation curves with very small amplitudes can be strongly affected by noise. In the present study, this small amplitude was reflected by low CNR values (0.34 ± 0.21), whereas these values usually exceed 10 under normal perfusion conditions. With such a low CNR, small fluctuations in the tissue attenuation curve caused by image noise could be mistaken for a slight degree of blood flow.

Another possible cause of above zero values of CBF and CBV revealed in our study could be selection of ICA as a surrogate of VOF. The ICA was the only possible choice because of vestigial enhancement or entirely lacked enhancement of intracranial veins in brain dead patients. In CTP, the arterial contrast bolus is scaled to have the same area under the curve as the venous outflow to correct for partial volume effects (PVE). This scaling is based on the assumption that large veins are unaffected by PVE. Riordan et al. showed that ICA in patients with normal cerebral circulation is unaffected by PVE [15]; however, in our study mean contrast enhancement in ICA was relatively low −346 HU; therefore, PVE could not be corrected sufficiently. This might result in overestimation of perfusion parameters. Taken together, these data suggest that the values of CBF and CBV above zero observed in the present study should not be interpreted as an indicator of residual cerebral flow in brain dead patients. The issue surrounding the inability of the CTP analysis software to show CBF and CBV values of zero in tissue from patients with BD may be addressed with further improvements involving denoising techniques, the use of a deconvolution algorithm, or both; however, these confounding factors do not preclude the potential clinical utility of CTP in the diagnosis of BD.

### Limitations of the Study

The present study was limited to the evaluation of the sensitivity of CTP and CTA in the diagnosis of BD because only brain dead patients were included. Further studies evaluating the sensitivity and specificity of this technique involving patients who are comatose, have locked-in syndrome, or are diagnosed as minimally conscious or in a persistent vegetative state are warranted, including the evaluation of differentially calculated perfusion algorithms and use of alternative AIFs or reference vessels, such as the superficial temporal artery and vein.

## Conclusion

Whole brain CTP potentially may be a feasible and highly sensitive test for use in the diagnosis of BD. Performing CTP together with the commonly used CTA, especially if the CTA finding is negative (i. e. shows intracranial filling), could increase the sensitivity of CTA.
